# Changes of MODY signal pathway genes in the endoplasmic reticulum stress in INS-1-3 cells

**DOI:** 10.1371/journal.pone.0198614

**Published:** 2018-06-07

**Authors:** Yanan Dong, Shirui Li, Wenhui Zhao, Yanlei Wang, Tingting Ge, Jianzhong Xiao, Yukun Li

**Affiliations:** 1 Department of Endocrinology, The Third Hospital of Hebei Medical University, Shijiazhuang, Hebei Province, China; 2 Department of Endocrinology, China-Japan Friendship Hospital, Beijing, China; 3 Department of Endocrinology, Beijing Tsinghua Changgung Hospital, School of Clinical Medicine, Tsinghua University, Changping District, Beijing, China; Universite Paris Diderot-Paris7 - Batiment des Grands Moulins, FRANCE

## Abstract

**Objective:**

Metabolic disturbances induce endoplasmic reticulum stress (ERS) in pancreatic beta cells. This study aims to investigate whether a common pathway exists in the ERS induced by various chemicals, including high levels of glucose and palmitate in INS-1-3 cells.

**Method:**

ERS in INS-1-3 cells was induced by exposure cells to thapsigargin (TG), tunicamycin (TM) or palmitic acid (PA) +high glucose (HG). Digital gene expression (DGE) profiling technique was used to detect differentially expressed genes. The profile of gene expression was detected by gene oncology (GO) function and pathway enrichment analysis. Nkx6.1 over-expression was established in INS-1-3 cell lines by lentivirus infection to revert the inhibition of Nkx6.1 expression found in the situation of ERS. Real time reverse transcription polymerase chain reaction (RT-PCR) was used to verify the expression changes of key genes. Cell viability was measured by 3-(4, 5-dimethylthiazol-2-yl)-2, 5-diphenyltetrazolium bromide (MTT) assay. The apoptosis was determined by flow cytometry. INS-1-3 cell function was measured by glucose stimulated insulin secretion test(GSIS).

**Results:**

As compared to control, DGE demonstrated that there were 135, 57 and 74 differentially expressed genes in TM, TG and HG+PA groups, respectively. Those differentially expressed genes were enriched to ERS, antigen processing and presentation, protein export pathways, and interestingly, the maturity onset diabetes of the young (MODY) pathway. Nkx6.1 is one of common down-regulated gene in MODY signaling pathway among TM, TG and HG+PA groups. Over-expression of Nkx6.1 ameliorated glucolipotoxicity induced apoptosis rate by 45.4%, and increased proliferation by 40.9%. At the same time, GSIS increased by 1.82 folds.

**Conclusions:**

MODY pathway genes expression was changed in the state of ERS. Over-expression of Nkx6.1 protected the INS-1-3 cells from glucolipotoxicity.

## Introduction

Ample evidences indicate that β-cell dysfunction determines the development and progression of type 2 diabetes (T2DM). By using homeostasis model assessment (HOMA), UK Prospective Diabetes Study (UKPDS) finds that pancreatic β-cell function have already lost by 50% at the time of diagnosis and declined by ∽5% annually[1]. The function failure of β-cells is associated with worsening of metabolic control, which is the major underlying mechanism for the acute and chronic complications in type 2 diabetes[2,3]. The preservation and even regaining of β-cell function is a critical therapeutic strategy for T2DM.

Pancreatic β cells have a highly developed endoplasmic reticulum system. It is crucial to their insulin secretion role to meet metabolic requirement. In fact, pancreatic β cells are one of the most vulnerable cells to ER stress among various tissues and cells. The failure or dysfunction of pancreatic β-cell, at least partially, is caused by irreversible damage of ER stress, but the underlying molecular mechanism is to be clarified. In previous studies, glucolipotoxicity induces ER stress both in vitro and in vivo and cause β cell dysfunction and plays an important role in development and progression of T2DM[4–6].

In this study, we hypothesized that a common pathway existed in β cells during ERS induced by various chemicals. We demonstrated that under the condition of ER stress, the expression changes of transcriptional factors related in MODY signaling pathway were associated with the impaired function of pancreatic β cells. We found that down regulation of Nkx6.1 expression was associated with the ER stress, and over-expression of Nkx6.1 showed a protective effect against glucolipotoxicity in INS-1-3 cells.

## Materials and methods

### Cells and culture

INS-1-3 cell line[7](a well-established insulin-secreting subclone cell line of INS-1) was obtained from the Institute of Clinical Medical Sciences, China-Japan Friendship Hospital. The cells were cultured in RPMI 1640 (GIBCO) medium (11.2 mM glucose) supplemented with 10% fetal calf serum, 50 μM 2-mercaptoethanol and 10^5^ U/L penicillin and 100 mg/L streptomycin and maintained in 5% CO2 at 37°C[8]. Normal cultured INS-1-3 cells were used as a control. Tunicamycin (TM, 2.5μg/mL), thapsigargin (TG, 0.1μmol/L) or palmitic acid (0.3mM) plus high glucose(16.7mM) was supplemented to induce ER stress.

### Preparation of sequencing libraries

The procedures including preparation of sequencing libraries, screening of differentially expression genes, gene oncology analysis and pathway analysis were performed at the Beijing Genome Institute (BGI) (Shenzhen, China). Briefly, the total RNA was extracted from INS-1-3 cells by using Trizol Reagent (Invitrogen, Carlsbad, CA). The mRNA was purified with DNase I and enriched with magnetic beads according to the manufacturer’s instructions. Then the fragmentation buffer (Ambion, Austin, TX) was added. Taken the fragmented mRNA as the template, first-strand cDNA was synthesized, followed by adding dNTPs, RNase H and DNA polymerase I to synthesize the second-strand cDNA. Double-stranded cDNAs were repaired and added an adenine base to the end. After amplification by PCR for fifteen cycles, the fragments were submitted to Agilent 2100 Bio-analyzer (Agilent Technologies, Palo Alto, CA, USA). Raw data were generated by using Illumina HiSeq 2000. Data filtering is performed to remove low quality reads or adaptor sequences, and clean reads were obtained.

### Screening of differentially expression genes

Clean reads were used to screen differentially expressed genes between groups. Fold changes (M) and absolute change values (D) were calculated. All M/D values were pooled together to generate the noise distribution. We applied NOIseq method to screen differentially expressed genes between two groups.

### Gene ontology enrichment analysis and pathway analysis

Gene ontology is a standardized gene functional classification system which contains three domains: cellular component, molecular function and biological process. Firstly, differentially expressed genes were mapped to GO terms in the database (http://www.geneontology.org/). Then significantly enriched GO terms were found by using hypergeometric test.

In order to understand biological functions of differentially expressed genes, we performed KEGG pathway analysis. This method firstly maps all differentially expressed genes to KEGG terms in the database (http://www.genome.jp/kegg/pathway.html). Then significantly enriched pathways were obtained by using calculating formula.

### Transfection

For the overexpression analysis of Nkx6.1 gene, INS-1-3 cells were cultured in a 24-well plate and were allowed to adhere for 24 hours. Plenti-III-EF1alpha vectors contained green fluorescent protein (GFP) or Nkx6.1 cDNA was being packed into lentiviruses and subjected to transfect INS-1-3 cells.

Transfected cells were cultured for 24 hours in complete medium containing polybrene. Then culture medium was replaced with complete medium. The cells stably expressing target gene were isolated by density gradient centrifugation with puromycin. Thirty hours after transfection, GFP expression was examined by flow cytometry.

### Quantitative reverse-transcription polymerase chain reaction (RT-PCR)

The total RNA (1 μg) was then reverse-transcribed to cDNA by using the GoScript™ Reverse Transcription System (Promega) according to the manufacturer’s instructions. For each reaction, cDNA was subjected to PCR by mixing with 19 μL of qPCR Master Mix (Go Taq^®^). The mRNA levels for the target genes were determined by real-time RT-PCR using the ABI PRISM 7500 Sequence Detection System (Foster City, CA). The threshold cycle (Ct) of each target gene was normalized using the Ct of β-actin as an internal control. The comparative 2^-ΔΔCt^ method was applied to calculate the relative expression of target gene in each sample relative to the control. The relative gene expression values were normalized to cells incubated in normal medium. The sequences of the specifically designed primers are as follows: β-actin (forward: 5’-TCAGGTCATCACTATCGGCAA-3’, reverse: 5’-TTACGGATGTCAACGTCACAC-3’), Nkx6.1 (forward: 5’-TACCCCCCATCAAGGATCCATT-3’, reverse: 5’-AGCCGCGTGCTTCTTCCTCCA-3’), Bhlha15 (forward: 5’-CTCGGATCCCCAGTCGGAAG-3’, reverse: 5’-CTCCGAAGACCCTTGGTCAC-3’), Bip/GRP78 (forward: 5’-TACCCCAGATTGAAGTCACCT-3’, reverse: 5’-TTCTCGGCGTCATTGACCA -3’), and CHOP (forward: 5’-ATCTCATCCCCAGGAAACGAA-3’, reverse: 5’-AGCCATAGAACTCTGACTGGA-3’).

### Western blot analysis

Proteins were subjected to SDS-PAG and transferred to nitrocellulose membranes. The membranes were blocked with 5% non-fat milk in TBST (10 mmol/L Tris, 150 mmol/L NaCl, and 0.1% Tween 20) for 2h and then incubated with the specific primary antibody of Nkx6.1(Santa Cruz, Dallas, TX, USA, 1:100), GRP78(Santa Cruz, Dallas, TX, USA, 1:100), CHOP(Santa Cruz, Dallas, TX, USA, 1:100) and tubulin (ComWin Biotech, CWBIO, 1:1000) over night at 4°C. Following incubation with secondary antibody, the bands were detected with the enhanced chemiluminescence (ECL) method. Immunoblots were scanned and quantified using Quantity One gel Analysis software.

### Cell viability

INS-1-3 cells were seeded into 96-well culture plates at 2×10^4^ cells/well. 24 hours after seeding, 0.3mM palmitic acid and 16.7mM glucose was supplemented. After incubation for 24 hours, 3-(4, 5-dimethylthiazol-2-yl)-2, 5-diphenyltetrazolium bromide (MTT) was added to the medium. Plates were incubated at 37°C for 4 hours. The resulting formazan crystals were solubilized in 150 μL of dimethyl sulfoxide(DMSO), and the optical density was read to assess the cell viability on a Microplate Reader (Bio-Rad iMark™, United States) with a test wavelength of 490nm.

### Cell apoptosis

The INS-1-3 cell apoptosis was detected by flow cytometry analysis using an Annexin V-fluorescein isothiocyanate (FITC)/propidium iodide (PI) kit (BD Pharmingen™, United States). Briefly, INS-1-3 cells seeded in 6-well plates (2×10^5^ cells/well) were incubated for 24 hours with and without palmitic acid (0.3mM) plus glucose(16.7mM). At the end of treatment, cells were harvested by trypsin-EDTA digestion. After washing with PBS, cells were re-suspended in binding buffer and incubated with 5 μL of Annexin V-FITC and PI solution for 15 min at room temperature in the dark. They were analyzed by flow cytometry (Beckman Coulter, Fullerton, CA, USA).

### Glucose stimulated insulin secretion (GSIS)

INS-1-3 cells were seeded at a density of 2×10^5^ cells/well in a 6-well plate. 24 hours after seeding, palmitic acid (0.3mM) plus glucose (16.7mM) was added to the cultures. After further 24 hours culture, medium was discarded. The cells were then rinsed twice with HEPES-buffered Krebs Ringer Bicarbonate solution (KRBH buffer: 128.8mM NaCl, 4.8mM KCl, 1.2mM KH_2_PO_4_, 1.2mM MgSO_4_, 2.5mM CaCl_2_, 5mM NaHCO_3_, and 10mM HEPES, pH 7.4), followed by pre-incubation in KRBH buffer containing 2.5mM glucose at 37°C for 30 minutes. After discarding of the pre-incubation buffer, the cells were then washed once with KRBH and incubated with 1mL of KRBH (2.5mM glucose; basal) or KRBH (20mM glucose; stimulated) for 30 minutes at 37°C. After incubation, supernatants from each well were collected. To measure intracellular insulin content, cells were washed once with phosphate-buffered saline, and acid-ethanol solution was added to each well, followed by sonication on ice. Intracellular insulin was extracted by separation of supernatant by centrifugation at 12,000 rpm×5 min at 4°C. The insulin concentration was measured using a rat insulin ELISA (ALPCO^®^) according to the manufacturer’s instruction.

### Statistical analysis

All the experiments were repeated 3 times. Data were presented as means ± standard deviation (SD), analyzed by the paired Student t test or by ANOVA, and *P*<0.05 was considered significant.

## Results

It is well known that ER stress of pancreatic β cells often occurs in the development of diabetes. Little is known about the profile of gene expression to various ER stress. DGE helps to screen the differentially expressed genes and pathway-based analysis helps to further understand genes biological functions. In this study, we used pathway enrichment analysis to identify significantly enriched metabolic pathways or signal transduction pathways under the whole genome background.

### 1. Digital gene expression (DGE) and gene oncology (GO) enrichment analysis

Digital gene expression (DGE) technology is an efficient and economic choice for analyzing gene expression. The original image data produced by the sequencer is transferred into sequences, which are defined as “raw reads”. As the raw reads may contain low quality reads or adaptor sequences, data filtering performed to obtain “clean reads” is necessary before starting further analysis. Digital gene expression(DGE) analysis showed that a total of 12476635, 12572138, 12227268 in TM groups, 12227268, 12048856, 12421592 in TG groups and 12572491, 12148256, 12074808 in HG+PA groups raw reads were generated using base calling. After removing of low quality sequences, the proportions of clean tags were >99% showing high sequencing quality.

We applied NOIseq method to screen differentially expressed genes between control and other three groups (TM, TG and HG+PA groups). Changes were classified according to absolute log2-fold changes in up- or down-regulation. Genes with small changes in expression (absolute log2-fold<1) were discarded. Numbers of up- and down-regulated genes from TM to control were 99 and 36. There were 45 and 12 in the TG group and 25 and 49 in HG+PA group, respectively.

To understand the distribution of gene functions of the species from the macro level, the genes were aligned to GO database. Compared with control, gene expressions had significant been enriched in 30 annotations in TM group (12 cellular component annotations, 6 molecular function annotations and 12 biological process annotations) and 28 annotations in TG groups (12 cellular component annotations, 5 molecular function annotations and 11 biological process annotations). And in HG+PA group, 16 annotations were closely related to the set of the imported genes. Among TM, TG and HG+PA groups, differentially expressed genes were most significantly enriched in ER components functional annotations (*P*<0.05).

**Fig 1:**
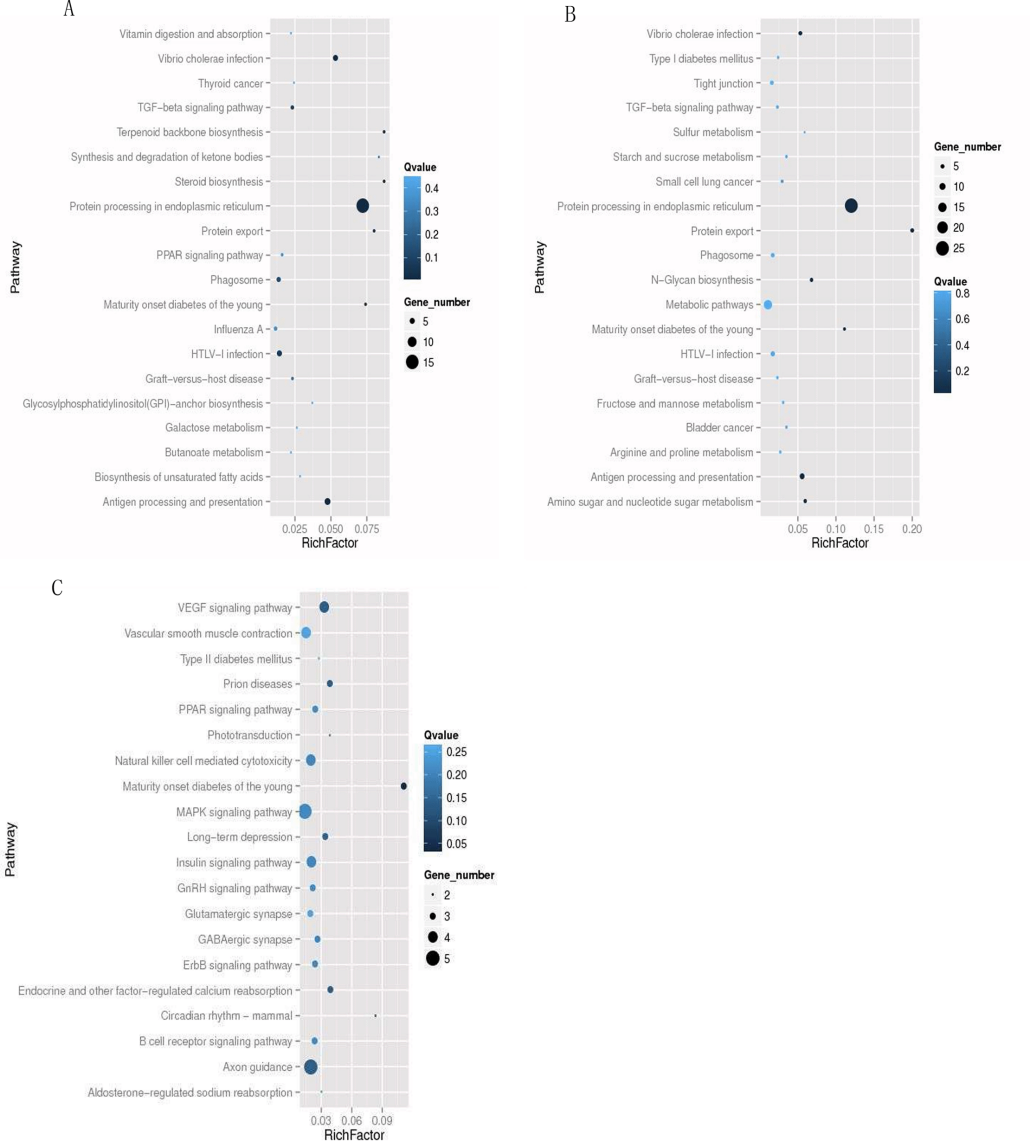
Statistics of pathway enrichment. A Statistic of Pathway Enrichment for con Vs TG, B Statistic of Pathway Enrichment for con Vs TG, C Statistic of Pathway Enrichment for con Vs HG+PA.

Next, we performed pathway enrichment analysis to understand the biological functions of altered genes. Based on the latest Kyoto Encyclopedia of Genes and Genomes (KEGG), the pathway enrichment analysis indicated that in TG group, the 57 genes were associated with 72 different pathways. And among them, 12 pathways were significant enrichment (Table 1, Fig 1A). While the 135 differentially expressed genes in TM group were associated with 120 pathways (Table 1, Fig 1B). Whereas in HG+PA group, 123 pathways were related to the altered expression genes, among them, 21 pathways were significant enriched (Table 1, Fig 1C). The identified pathways were related with endoplasmic reticulum stress, antigen processing and presentation, protein export, and most of all, the maturity onset diabetes of the young (MODY) pathway. By comparing MODY signaling pathway of TM, TG and HG+PA groups, we found a series of common abnormal-expressed genes. In all the three groups, motor neuron and pancreas homeobox 1 (Mnx1) declined to 0.45, 0.59, 0.35, Nk6 homeobox 1 (Nkx6.1) declined to 0.55, 0.46, 0.54, respectively, while the basic helix-loop-helix family member a15 (Bhlha15) rose to 3.84 and 3.7 in TM and TG groups.

**Table 1:** Statistics of pathway enrichment.

Enrichment Pathway(ID)	Q value*(TM)	Q value*(TG)	Q value*(HG+PA)
protein processing in endoplasmic reticulum(ko04141)	<0.001	<0.001	≥0.05
protein export(ko03060)	<0.001	0.041	≥0.05
antigen processing and presentation(ko04612)	0.003	<0.001	≥0.05
Vibrio cholerae infection(ko05110)	0.025	<0.001	≥0.05
N-Glycan biosynthesis(ko05110)	0.025	≥0.05	≥0.05
mature onset diabetes of the young(ko04950)	0.025	0.041	0.032
amino sugar and nucleotide sugar metabolism(ko00520)	0.034	≥0.05	≥0.05
Terpenoid backbone biosynthesis(ko00900)	≥0.05	0.041	≥0.05
steroid biosynthesis(ko00100)	≥0.05	0.041	≥0.05

* Note: pathways with Q value<0.05 are significantly enriched in DEGs

**Fig 2:**
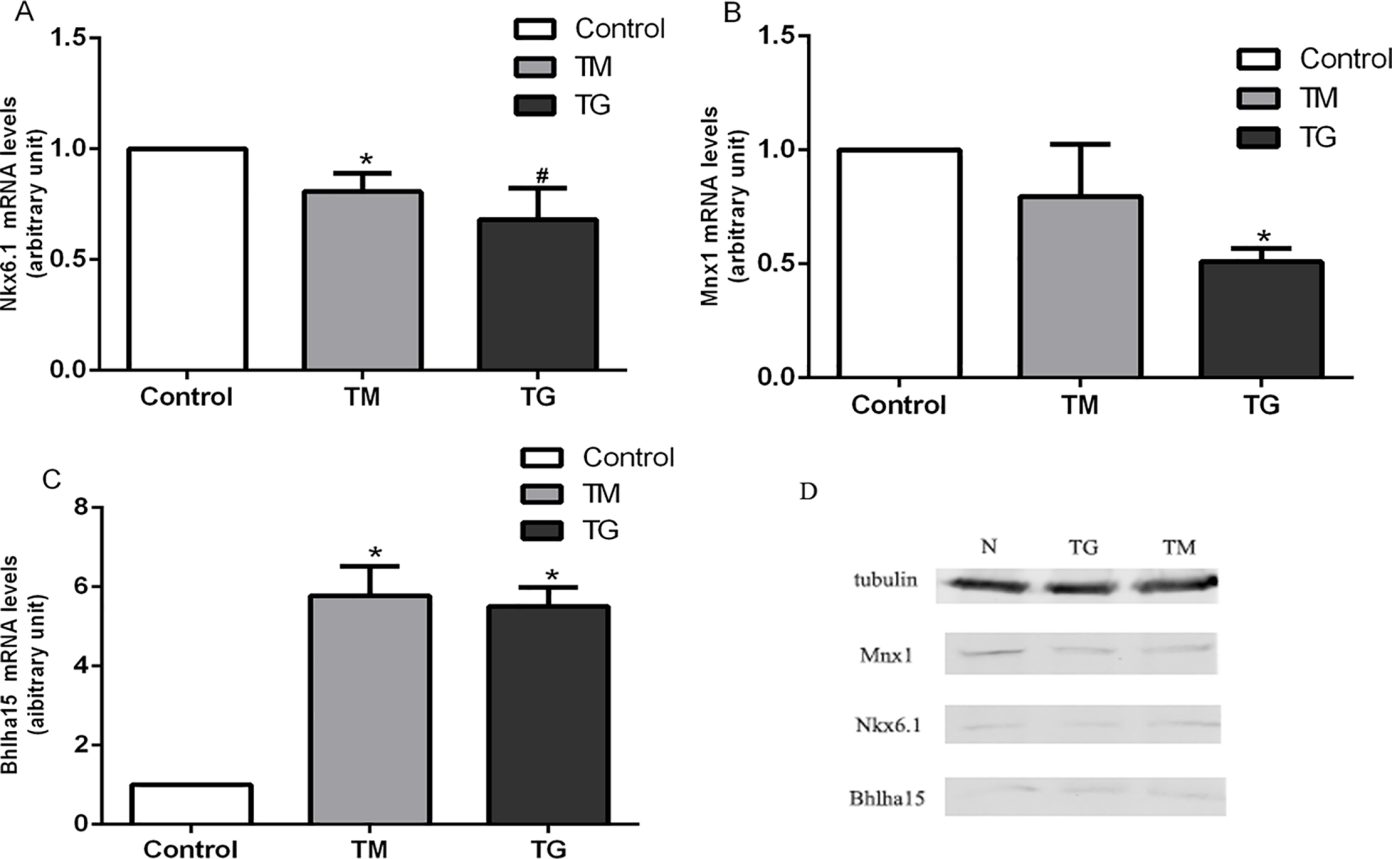
A quantitative analysis of Nkx6.1, Mnx1 and Bhlha15 mRNA levels. *P<0.05 compared to control, ^#^*P*<0.01 compared to control.

We performed RT-PCR and WB to confirm some key findings of the DEG and GO analysis. We used RT-PCR and WB to measure the mRNA and protein levels of 3 altered expression genes in MODY signaling pathway. The expressions of Nkx6.1, Mnx1 and Bhlha15 genes were confirmed by RT-PCR (Fig 2).

These data suggested that ER stress modulated MODY signal pathway genes in INS-1-3 cells.

### 2. The apoptosis and proliferation of INS-1-3 cells

**Fig 3:**
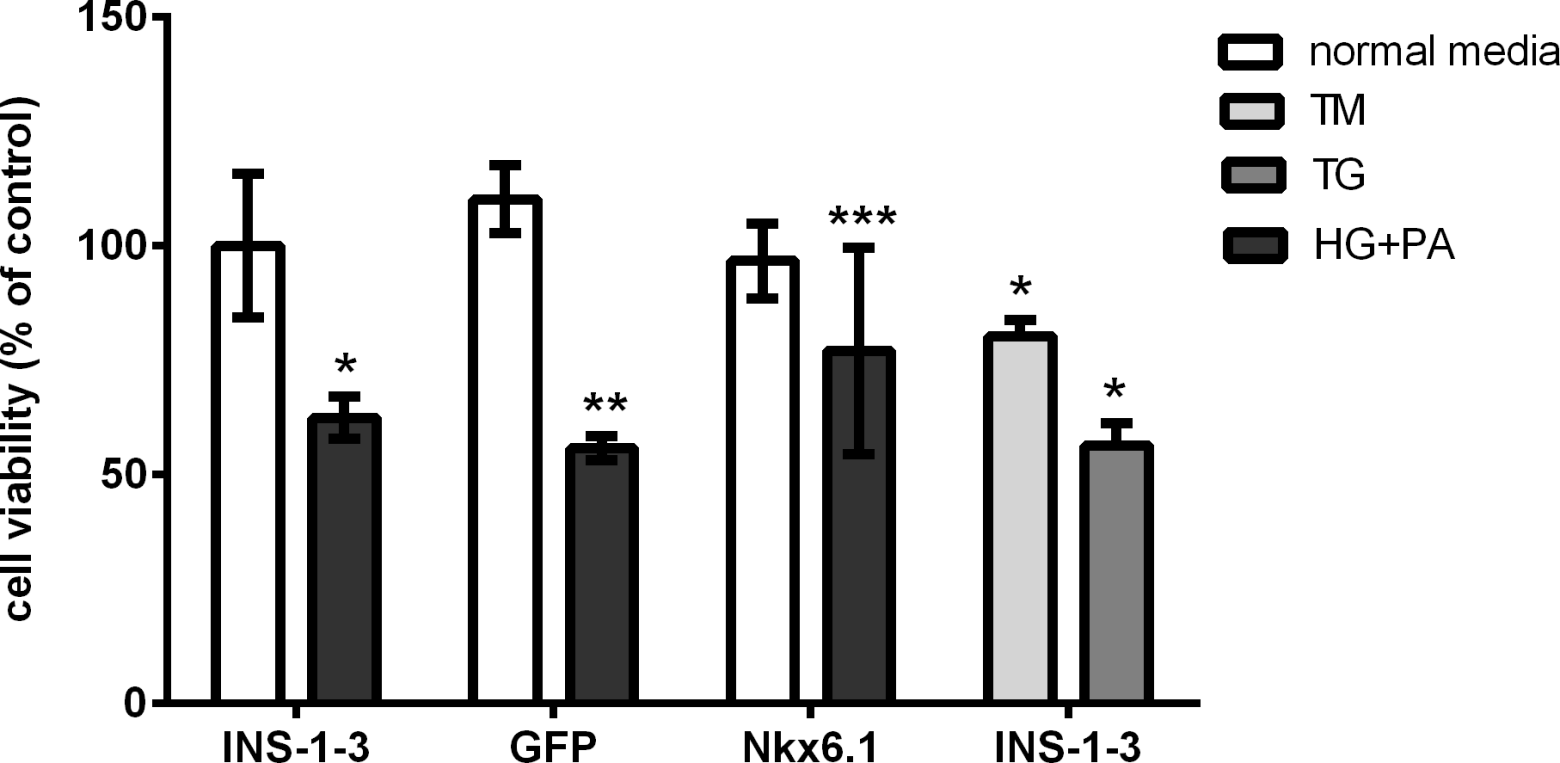
Cell viability in INS-1-3 cells. ERS was induced by exposure cells to thapsigargin (TG, 0.1μmol/L), tunicamycin (TM, 2.5μg/mL) or palmitic acid (PA, 0.3mmol/L) +high glucose (HG, 16.7 mmol/L). Cells viability was measured by MTT assay. *P<0.05 compared with INS-1-3 (normal medium), **P<0.01 compared with GFP (normal medium), ***P>0.05 compared with Nkx6.1 (normal medium), ***P<0.05 compared with GFP(HG+PA) and Nkx6.1 (HG+PA).

In the light of the importance of Nkx6.1 gene in the development of pancreatic β cells and the suppression of Nkx6.1 gene to ER stress, overexpression of Nkx6.1 might attenuate the ERS induced by glucolipotoxicity. To test this speculation, we measured the INS-1-3 cell viability by using MTT with different experimental manipulations. There were no significant differences among INS-1-3 cells, GFP-expressing and Nkx6.1-expressing INS-1-3 cells incubated in normal culture medium. As the cells exposed to high glucose plus palmitic acid, the proliferation of both INS-1-3 cells and GFP-expressing cells decreased by about 45% and 50%, respectively (*P*<0.01). In contrast, treatment with high glucose plus palmitic acid did not significantly inhibit the cell proliferation in Nkx6.1 over-expressing cells (Fig 3, *P*>0.05). It suggested overexpression of Nkx6.1 gene could alleviate impairment induced by chronic exposure to elevated concentrations of glucose and palmitic acid.

**Fig 4:**
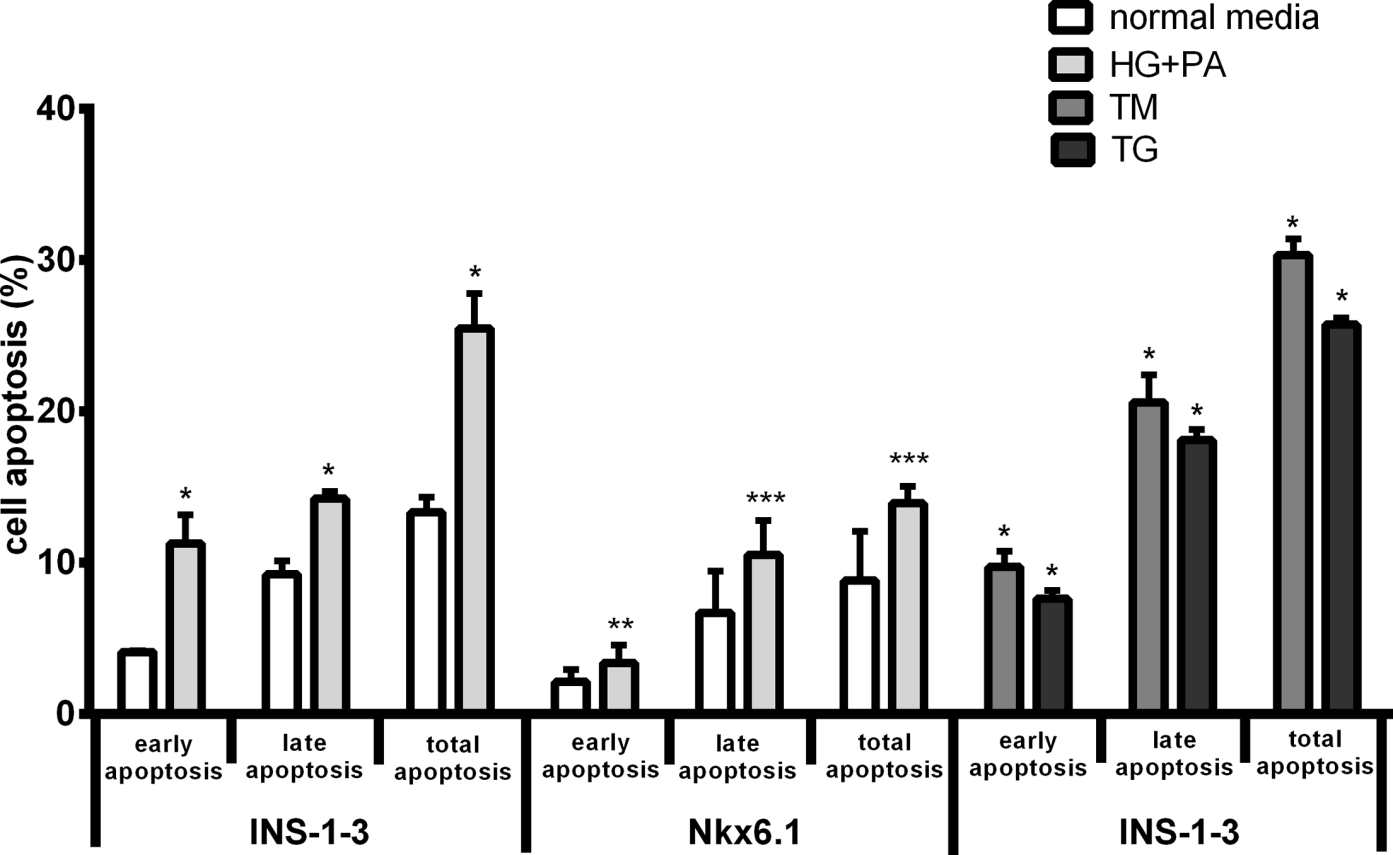
The apoptosis of INS-1-3 cells. ERS was induced by exposure cells to thapsigargin (TG, 0.1μmol/L), tunicamycin (TM, 2.5μg/mL) or palmitic acid (PA, 0.3mmol/L) +high glucose (HG, 16.7 mmol/L). The degree of apoptosis was detected by flow cytometry. *P<0.01 compared with INS-1-3 (normal medium). **P>0.05 compared with Nkx6.1(normal medium, early apoptosis), ***P<0.01 compared with Nkx6.1 (HG+PA).**^,^***P<0.01 compared with INS-1-3(HG+PA).

We next explored the role of Nkx6.1 gene in maintaining cell viability. The flow cytometry analysis showed that there were no significant differences of apoptosis markers between Nkx6.1-expressing cells and un-transfected cells in normal culture medium. After 24 hours exposure to high level of glucose plus palmitic acid, the levels of marker for early apoptosis, late apoptosis and total apoptosis were significantly elevated by 174.9%, 54.2% and 91.3% in INS-1-3 cells, respectively (Fig 4, *P*<0.05). In contrast, exposure Nkx6.1 over-expressing INS-1-3 cells to high glucose plus palmitic acid did not change the early and late stage of apoptosis markers (*P*>0.05). Consequently, these apoptosis markers significantly increased in INS-1-3 cells compared to Nkx6.1 expressing INS-1-3 cells after exposure to in HG plus PA (*P*<0.01). These data suggested that Nkx6.1 played a protective role towards glucolipotoxicity.

### 3. Nkx6.1 over-expression attenuated the impairment of insulin secretion in glucolipotoxicity in INS-1-3 cells

**Fig 5:**
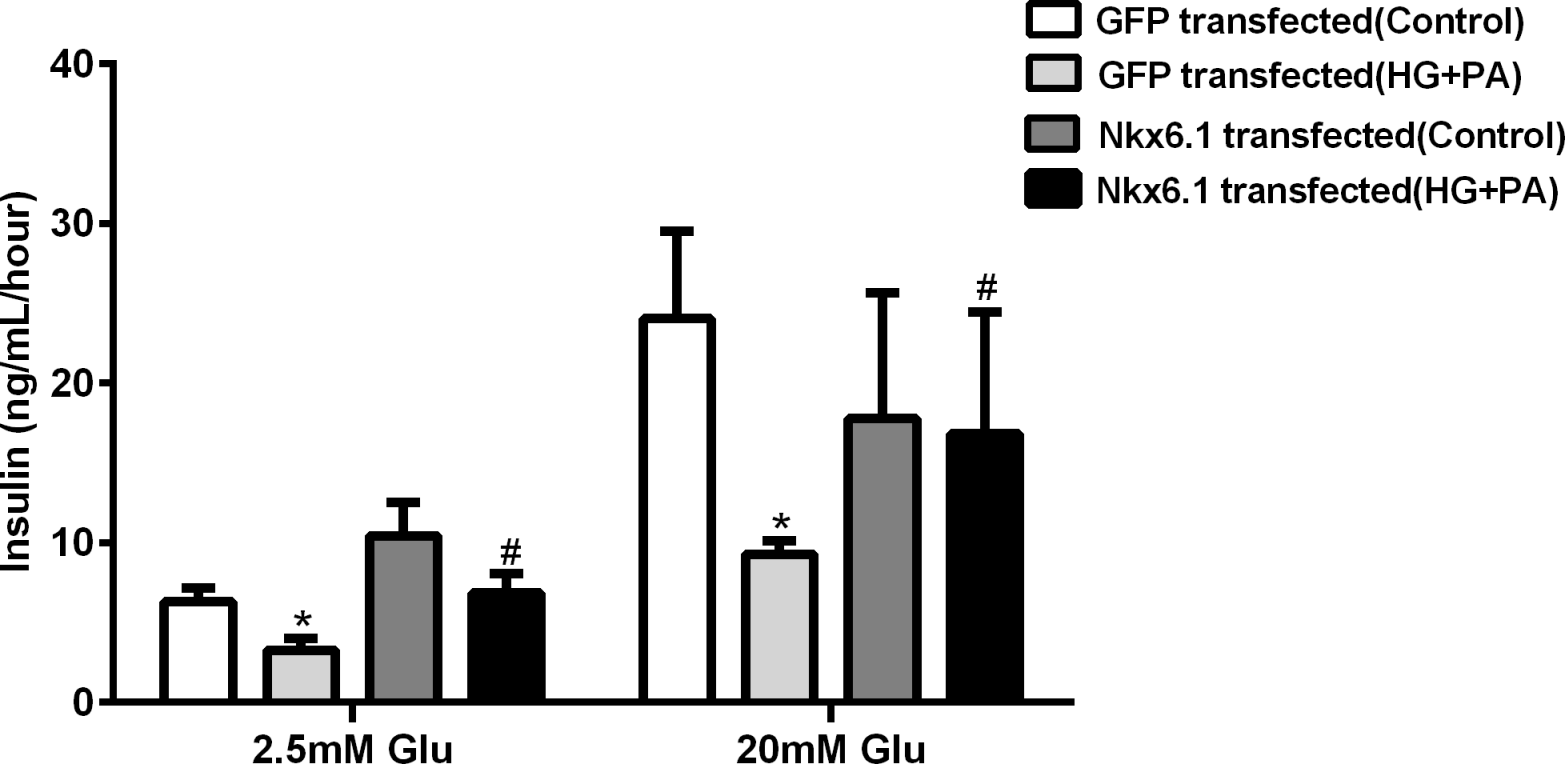
Glucose stimulated insulin secretion of INS-1-3 cells. Cells were incubated with normal medium as control, 16.7mM glucose plus 0.3mM palmitic acid (HG+PA) was added to induce ERS. Insulin secretion was detected by ELISA induced by 20mM glucose as GSIS. *P<0.05 compared with GFP (normal medium). ^#^*P*>0.05 compared with Nkx6.1 (normal medium).

Overexpressing Nkx6.1 was used to investigate the impact of Nkx6.1 on insulin secretion of INS-1-3 cells. As shown in Fig 5, the basal insulin secretion (BIS) and the glucose stimulated insulin secretion (GSIS) were not different between Nkx6.1 over-expressing and GFP expressing INS-1-3 cells (*P*>0.05) in normal culture. After chronic exposure GFP expressing cells to high levels of glucose plus palmitic acid, BIS and GSIS decreased by 32.3% and 56.0%, respectively (*P*<0.05). Nkx6.1 over-expressing group demonstrated only a 5.8% decrease of GSIS by (*P*>0.05), It suggested over expression of Nkx6.1 protected INS-1-3 cells from glucolipotoxicity.

### 4. Glucolipotoxicity induced similar changes of ERS marker as TG and TM in INS-1-3 cells

**Fig 6:**
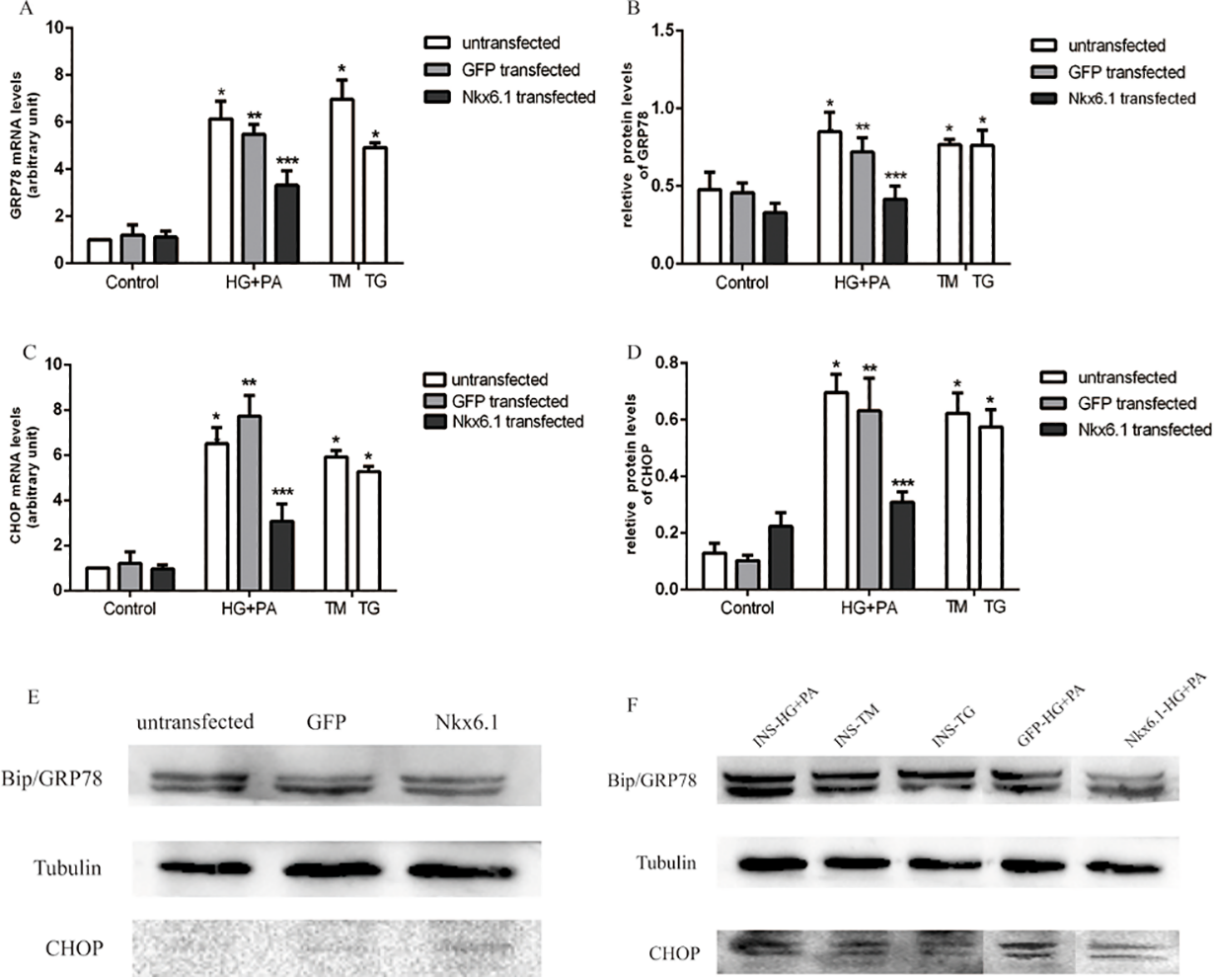
mRNA and protein levels of GRP78 and CHOP in INS-1-3 cells. ERS was induced by exposure cells to thapsigargin (TG, 0.1μmol/L), tunicamycin (TM, 2.5μg/mL) or palmitic acid (PA, 0.3mmol/L) +high glucose (HG, 16.7 mmol/L). (A) A real time reverse transcription polymerase chain reaction was used to measure GRP78 mRNA levels. *P<0.01 compared with INS-1-3 (normal medium). **P<0.01 compared with GFP(normal medium). ***P<0.01 compared with Nkx6.1(normal medium), INS-1-3 (TM, TG, HG+PA) and GFP(HG+PA). (B) The protein levels of GRP78 were measured by Western blot. Tubulin was used as loading control. *P<0.01 compared with INS-1-3 (normal medium). **P<0.01 compared with GFP(normal medium). ***P>0.05 compared with Nkx6.1(normal medium). ***P<0.01 compared with INS-1-3 (TM, TG, HG+PA) and GFP(HG+PA). (C) A Real time reverse transcription polymerase chain reaction was used to measure CHOP mRNA levels. *P<0.01 compared with INS-1-3 (normal medium). **P<0.01 compared with GFP(normal medium). ***P<0.01 compared with Nkx6.1(normal medium), INS-1-3 (TM, TG, HG+PA) and GFP(HG+PA). (D) The protein levels of CHOP were measured by Western blot. Tubulin was used as loading control. *P<0.01 compared with INS-1-3 (normal medium). **P<0.01 compared with GFP(normal medium). ***P>0.05 compared with Nkx6.1(normal medium). ***P<0.01 compared with INS-1-3 (TM, TG, HG+PA) and GFP(HG+PA). (E) Western Blot analysis for GRP78 protein levels. (F) Western Blot analysis for CHOP protein levels.

Exposure to TM and TG are known to induce ER stress. To examine the similarity between glucolipotoxicity and other ER stress inducers, the mRNA and protein levels of ER stress markers, Bip/GRP78 and CHOP were analyzed [9]. After exposed to TM, TG, and HG plus PA, Bip/GRP78 mRNA levels were significantly increased by 4.92 folds, 6.96 folds and 6.14 folds respectively (Fig 6A, *P*<0.01). And the protein levels of Bip/GRP78 increaesd by 1.60 folds, 1.59 folds and 1.78 folds. Meanwhile, TM, TG and HG plus PA significantly increased CHOP mRNA and protein levels (Fig 6C and 6D, *P*<0.01). Therefore, glucoliopotoxicity could cause ER stress similar to TM and TG in INS-1-3 cells.

After exposure to high glucose plus palmitic acid, both mRNA levels and protein levels of Bip/GRP78 in Nkx6.1 over-expressing cells was found to be significantly lower than GFP expressing (60.5% and 57.7%) cells. And after over-expressing Nkx6.1 in INS-1-3 cells, mRNA levels and protein levels of CHOP were significant lower than GFP expressing INS-1-3 cells, indicating that over-expression of Nkx6.1 attenuated ERS in INS-1-3 cells.

### 5. Change Nkx6.1 expression in INS-1-3 cells

The ER stress lead to a down-regulation of Nkx6.1, we investigated whether Nkx6.1 overexpression protected from glucolipotoxicity.

**Fig 7:**
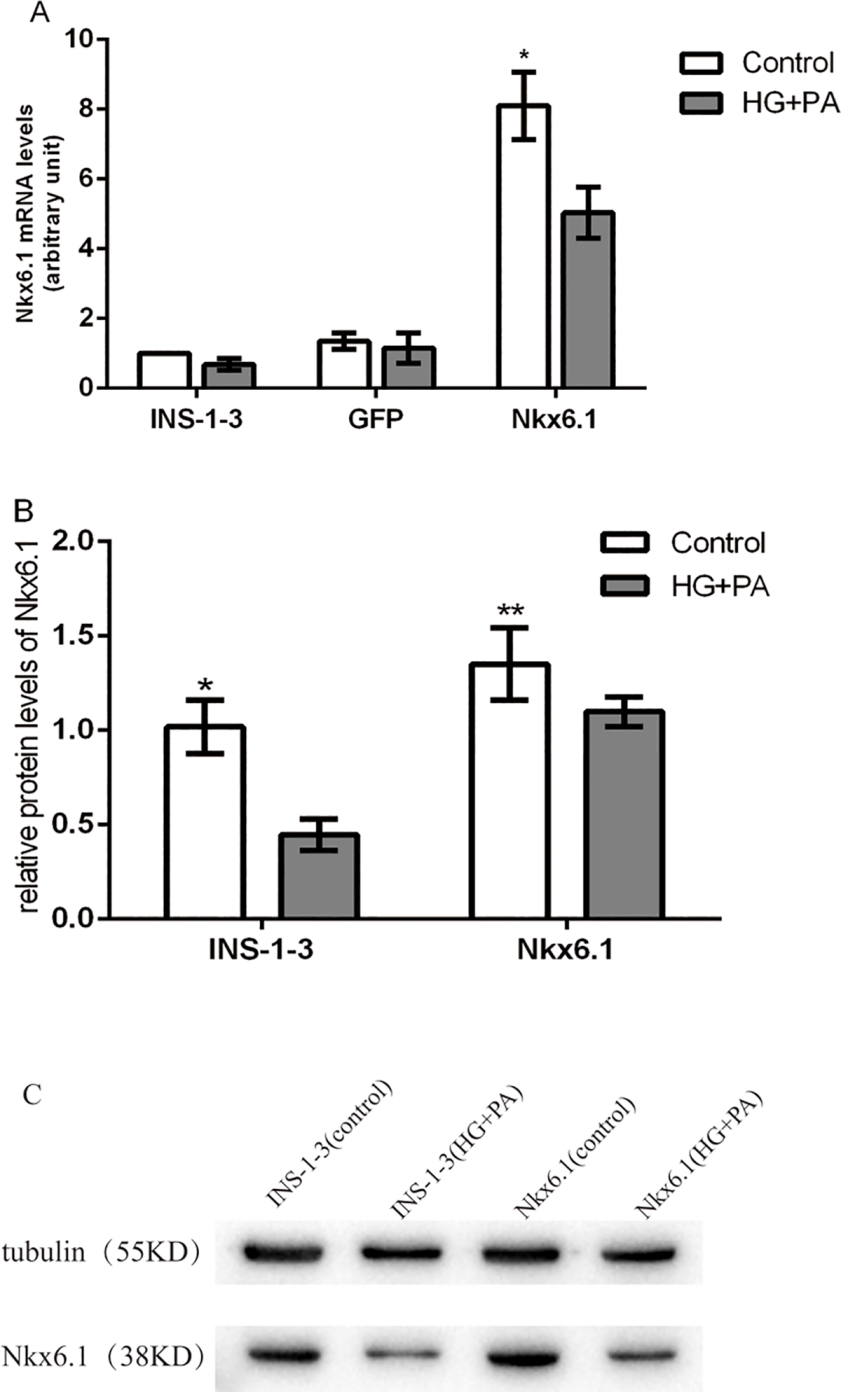
mRNA and protein levels of Nkx6.1 in INS-1-3 cells. ERS was induced by exposure cells to thapsigargin (TG, 0.1μmol/L), tunicamycin (TM, 2.5μg/mL) or palmitic acid (PA, 0.3mmol/L) +high glucose (HG, 16.7 mmol/L). (A) A Real time reverse transcription polymerase chain reaction was used to measure Nkx6.1 mRNA levels. *P<0.01 compared with control. (B) The protein levels of Nkx6.1 were measured by Western blotting. Tubulin was used as loading control. *P<0.01 compared with INS-1-3 (HG+PA), **P>0.05 compared with Nkx6.1 (HG+PA) (C) Western blotting analysis for Nkx6.1 protein levels.

The INS-1-3 cells treated with lentiviruses contained Nkx6.1 exhibited a significant increase in Nkx6.1 mRNA level at 8.1 folds and 6 folds relative to un-transfected cells and GFP-expressing cells (*P*<0.05). Meanwhile the Nkx6.1 protein was also significantly elevated after transfection (*P*<0.05), suggesting that stable gene transfection has been achieved in INS-1-3 cell line (Fig 7A).

Both Nkx6.1 mRNA and protein levels reduced in INS-1-3 and GFP cells treated to high concentration of glucose and palmitic acid for 24 hours, but the reduction attenuated in the Nkx6.1 over-expressing cells. Though Nkx6.1 protein level decreased after being exposed to HG and PA, it is still markedly elevated in the Nkx6.1 over-expressed cells compared to the control (Fig 7B).

## Discussion

The endoplasmic reticulum is the intracellular organelle of protein synthesis, folding and transport. ERS is defined as an imbalance between the folding capacity of the ER and the protein load, resulting in the accumulation of misfolded and unfolded protein. ERS can be induced by chemicals, high level of glucose and palmitate and other stress conditions in pancreatic β cells. Recent evidences suggest that ER stress may responsible for the molecular mechanism of glucolipotoxicity of β cells in type 2 diabetes[10–12]. Whether ERS induced by various conditions share common signal pathway remains to be clarified. In the context of association of metabolic disturbance in T2DM, gaining a better understanding of molecular mechanisms in ERS of beta cells induced by high levels of glucose and fatty acid is therefore of great relevance for development of new diabetes therapies.

TM and TG were chemicals known to induce ERS. Hyperlipidemia is very common in T2DM, so we applied high concentrations of glucose and palmitic acid to mimic pathophysiological state during diabetes.

In this study, there were 57 differentially expressed genes in TG group, 135 expressed genes in TM group and 74 differentially expressed genes in HG+PA group. By using functional and pathways enrichment method, we analyze the involved signal pathways among altered-expression genes. Not surprisingly, the profile of ERS markers under glucolipotoxicity was similar to that induced by TM or TG. Interestingly, 3 genes involved in MODY signaling pathway, including Bhlha15, Mnx1 and Nkx6.1, changed. It suggested the changed expression of genes in MODY signaling pathway maybe related to the impaired function of INS-1-3 cells in ERS state.

The homeobox transcription factor NK6 homeobox 1 (Nkx6.1) is a transcription factor that expressed in various cells and tissues. It was initially found in a hamster insulinoma cell cDNA library[13]. Studies in the mice have demonstrated that the exocrine, endocrine, and ductal lineages of the adult pancreas are characterized by the co-expression of a combination of transcription factors, including Pdx1, Ptf1a, Nkx6.1, Cpa, Myc, and Sox9[14–19]. And the expression of Nkx6.1 is required for development of the β cell lineage from endocrine progenitors[20]. Moreover, knock-down of Nkx6.1 in mice results in a >90% decrease of β-cell mass[21]. Over-expression of Nkx6.1 causes a clear enhancement of glucose stimulated insulin secretion (GSIS) and stimulation of cell proliferation in both human and rat islet β cells[22,23]. To explore the role of Nkx6.1 during glucolipotoxicity, we over-expressed Nkx6.1 in INS-1-3 cells by lentiviruses transduction. As shown in results, overexpression of Nkx6.1 decreased the levels of ERS markers in INS-1-3 cells in glucolipotoxicity. At the same time, Nkx6.1 over-expression alleviated the increased apoptosis, decreased proliferation and inhibited GSIS in INS-1-3 cells induced by glucolipotoxicity.

In summary, ERS changed the expression of MODY signal pathway genes. Overexpressing of Nkx6.1 gene could partially alleviate ERS in glucolipotoxicity. These findings highlight the regulation of Nkx6.1 may have potential role for the treatment of diabetes.

## Supporting information

S1 Fig Original uncropped and unadjusted blots. (A)(B) Original uncropped and unadjusted blots for Fig 1E and 1F. (C)(D) Original uncropped and unadjusted blots for Fig 4C. (E)(F) Original uncropped and unadjusted blots for Fig 3D.
(TIF)
