# Supplementary figures and images for: Changes of MODY signal pathway genes in the endoplasmic reticulum stress in INS-1-3 cells
Source: PLoS One. 2018 Jun 7;13(6):e0198614. doi: 10.1371/journal.pone.0198614 (PMC5991669; doi:10.1371/journal.pone.0198614)

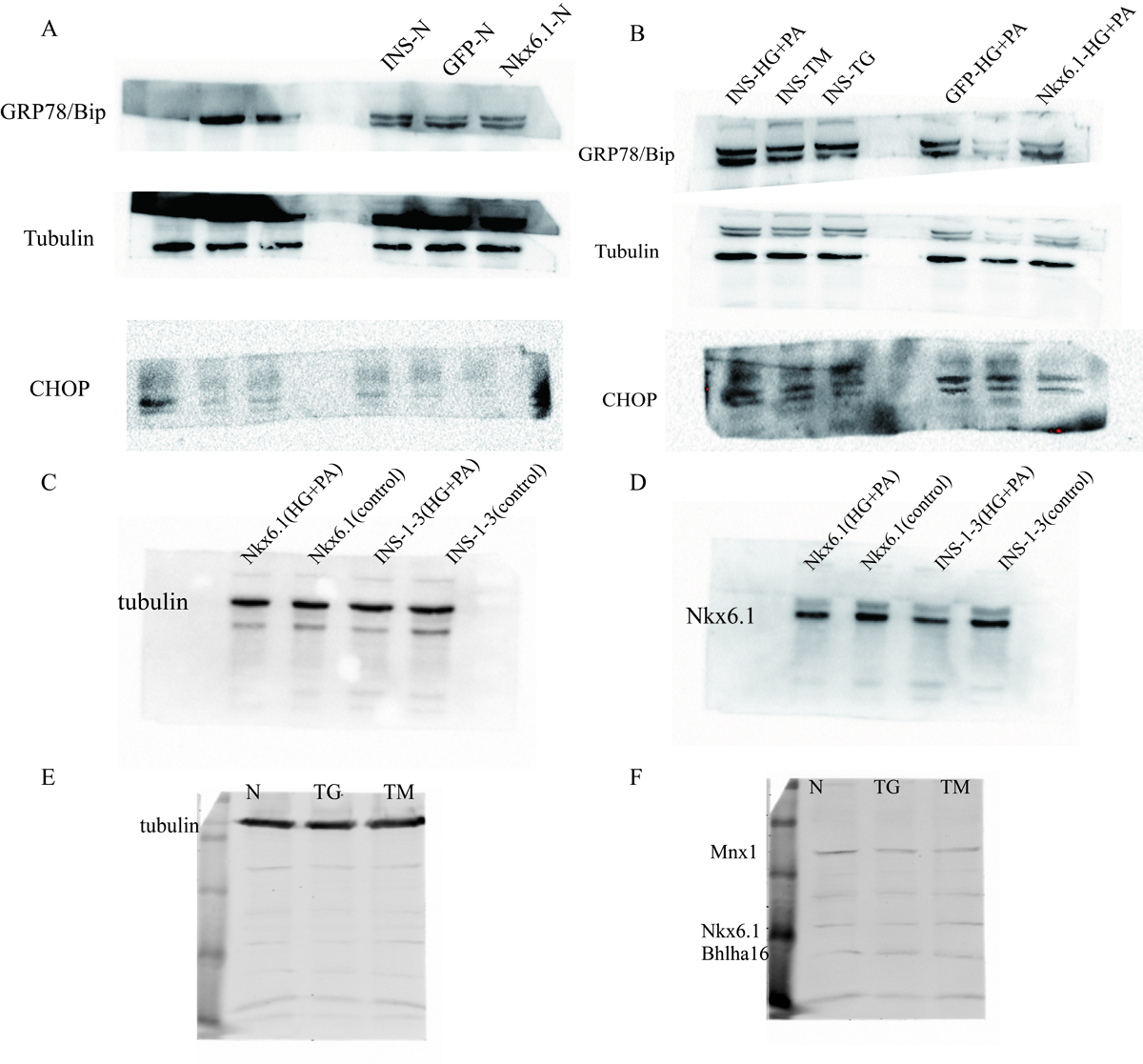

Supplement: S1 Fig — (A)(B) Original uncropped and unadjusted blots for Fig 1E and 1F. (C)(D) Original uncropped and unadjusted blots for Fig 4C. (E)(F) Original uncropped and unadjusted blots for Fig 3D. (TIF) [file pone.0198614.s001.tif]
